# Structural and Photophysical Properties of Methylammonium Lead Tribromide (MAPbBr_3_) Single Crystals

**DOI:** 10.1038/s41598-017-13571-1

**Published:** 2017-10-20

**Authors:** Kai-Hung Wang, Liang-Chen Li, Muthaiah Shellaiah, Kien Wen Sun

**Affiliations:** 1 Department of Applied Chemistry, National Chiao Tung University, 1001 University Road, Hsinchu, 30010 Taiwan; 2 Department of Electronics Engineering, National Chiao Tung University, 1001 University Road, Hsinchu, 30010 Taiwan; 3 Center for Nano Science and Technology, National Chiao Tung University, Hsinchu, 30010 Taiwan

## Abstract

The structural and photophysical characteristics of MAPbBr_3_ single crystals prepared using the inverse temperature crystallization method are evaluated using temperature-dependent X-ray diffraction (XRD) and optical spectroscopy. Contrary to previous research reports on perovskite materials, we study phase transitions in crystal lattice structures accompanied with changes in optical properties expand throughout a wide temperature range of 300–1.5 K. The XRD studies reveal several phase transitions occurred at ~210 K, ~145 K, and ~80 K, respectively. The coexistence of two different crystallographic phases was observed at a temperature below 145 K. The emission peaks in the PL spectra are all asymmetric in line shape with weak and broad shoulders near the absorption edges, which are attributed to the Br atom vacancy on the surface of the crystals. The time-resolved PL measurements reveal the effect of the desorption/adsorption of gas molecules on the crystal surface on the PL lifetimes. Raman spectroscopy results indicate the strong interplays between cations and different halide atoms. Lastly, no diamagnetic shift or split in emission peaks can be observed in the magneto-PL spectra even at an applied magnetic field up to 5 T and at a temperature as low as 1.5 K.

## Introduction

Methylammonium lead halide perovskite solar cells have become efficient and progressed faster than any other solar cells to date in a few years since their invention^1–4^. The remarkable performance of these solar cells has initiated the exploration of applications in optoelectronics, such as light-emitting diode^5^, lasers^6,7^, and photodetectors^8,9^. Organometal halide perovskites, particularly the CH_3_NH_3_PbX_3_ (X = Br, I, Cl), which show impressive properties for photovoltaic applications, are being widely studied. Their low in manufacturing cost and ease of preparation and production make them suitable candidates for future technologies. However, commercializing perovskite devices is impeded by rapid material degradation^10,11^, hysteresis^12,13^, and environmental factors, such as humidity and temperature^14^. The performance and stability of hybrid perovskite devices are strongly affected by defect states and film quality, which affect carrier lifetime^15–17^. Meanwhile, perovskite films produced with large grains show an improved crystalline quality^18–20^. Carrier diffusion length over 100 μm and high electron and hole mobilities of 2800 and 9400 cm^2^-V^−1^s^−1^, respectively, have been demonstrated in MAPbI_3_ and MAPbBr_3_ single crystal^21–23^.

We lack a fundamental understanding of the structural and photphysical properties of perovskite devices despite the rapid advances of these hybrid materials. So far, the unprecedented improvement in the power conversion efficiency of perovskite solar cells is due mostly to good film quality and proper design in device architecture and contacts. The electronic structures and optical properties of perovskite should be fully characterized to further optimize these devices because carrier generation, recombination, and transportation processes play important roles in optoelectronic and photovoltaic performance.

Early studies for characterizing the material properties of perovskites primarily focused on polycrystalline thin films^24–30^. However, the intrinsic nature of the electronic or optoelectronic properties of polycrystalline thin films may be overshadowed by the micro- and/or nanostructure quality and non-crystalline domains. On the contrary, the absence of grain boundaries and non-crystalline domains in single crystals makes them an ideal platform for studying the intrinsic material and optical properties of perovskite, and can therefore help improve perovskite polycrystalline thin film solar cells. Recently, the electronic structure and optical properties of α–phase CH_3_NH_3_PbBr_3_ bulk crystals at 0.73–6.45 eV were studied by spectroscopic ellipsometry^31^ and the crystal showed a strong optical transition at ~2.3 eV; in addition, the orientations of the cations in the crystal were randomly distributed at room temperature. Exceptionally long and balanced electron and hole diffusion lengths and low trap-state density were observed in sizable MAPbX_3_ single crystals^21,32^. The dynamic behavior of photocarriers, such as surface or bulk recombination kinetics and free-carrier diffusion, was reported using transient optical spectroscopy in CH_3_NH_3_PbBr_3_ single crystals^33–35^. Recently, Tilchin *et al*.^36^ deduced an exciton binding energy of 15.33 meV and a Bohr radius of ~4.38 nm at a low temperature in the orthorhombic phase of MAPbBr_3_ single crystals.

The efficiency of MAPbI_3_ perovskite solar cells has reached over 20%; however, a clear scenario in crystal/energy band structures and a mechanism for exciton separation and carrier transport are still missing. Therefore, the intrinsic properties of perovskite materials should be studied for future device refinement. For instance, there is still strong debating on the ferroelectricity^37–39^ of hybrid perovskite and no conclusive evidence has been reported so far. Despite diligent research, opinions regarding the aforementioned fundamental properties are contradicting in the literature, and further work is required to reconcile these conflicts.

Thus, MAPbX_3_ single crystals were prepared from a solution and showed less defects in material and grain boundaries. In the present study, we focus on MAPbBr_3_ single crystals to differentiate the intrinsic structural and photophysical properties between bulk materials and thin films. This study investigates these properties using single and powder x-ray diffraction (XRD) spectroscopy, steady-state and time-resolved photoluminescence (PL) spectroscopy, absorption/transmittance spectroscopy, Raman spectroscopy, and magneto-PL spectroscopy at temperatures of 300–1.5 K.

## Methods

### General Information

The structural properties and phase transitions of the as-grown MAPbBr_3_ single crystals were characterized by XRD. Temperature-dependent single-crystal and powder XRD studies were conducted at 300–20 K from a Bruker D8 Discover X-ray Diffraction System. Transmission electron microscopy (TEM) samples and high-resolution TEM (HRTEM) studies were done using a TESCAN LYRA 3 Dual-Beam Focus Ion Beam Microscope and a JEOL-JEM-2100F, respectively. Temperature-dependent absorption/transmittance measurements were taken at 300–30 K using a combination of a HOROBA iHR-320 spectrometer, Xenon lamp, and liquid-nitrogen cooled CCD detector with the samples placed in a cryostat. Temperature-dependent steady-state and transient PL spectra were obtained at 300–15 K with a HOROBA iHR-550 spectrometer, Si/PMT photodetector, and diode-pumped solid state laser operated at 405 nm. Raman interrogations were employed using a HOROBA and a Lab RAM HR instrumental set up using a 632 nm He-Neon laser.

The single crystal was attached to a sample holder with N-grease in Faraday geometry at the bottom of an insert equipped with a fiber probe to obtained magneto-PL measurements under a low temperature and high magnetic field. The insert was placed in a dilution refrigerator and cooled to 1.5 K. We performed the PL spectroscopy measurements through the fiber for abovementioned samples at an excitation wavelength of 405 nm with an applied magnetic field either parallel or perpendicular to the crystal (100) direction.

### Synthesis of MAPbBr_3_ single crystal

The MAPbBr_3_ single crystals were prepared from a solution using a slightly modified version of the inverse temperature crystallization (ITC) method reported in a previous study^22^. Lead bromide (PbBr_2_, 99.999%, Alfa Aesar), methylammonium bromide (CH_3_NH_3_Br, 98%, Sigma-Aldrich), dimethylformamide (DMF, 99.5%, Merck KGaA), and dimethyl sulfoxide (DMF, 99.7%, Sigma-Aldrich) were used as received and without further purification. A total of 0.2463 g of CH_3_NH_3_Br were added quickly to 2.2 mL DMF solution in an ultrasonic bath under a N_2_ atmosphere at room temperature for 5 min until the CH_3_NH_3_Br was totally dissolved. Then, 0.734 g PbBr_2_ was added to 2 mL CH_3_NH_3_Br/DMF solution and stirred for 15 min until the solution became transparent. The solution was filtered using PVDF filter. The filtrate was placed in a vial and kept in an oil bath undisturbed at 80 °C for 4 h. Millimeter-sized crystals were taken out of the vial once formed and dried with a nitrogen gun.

## Results and Discussions

**Figure 1:**
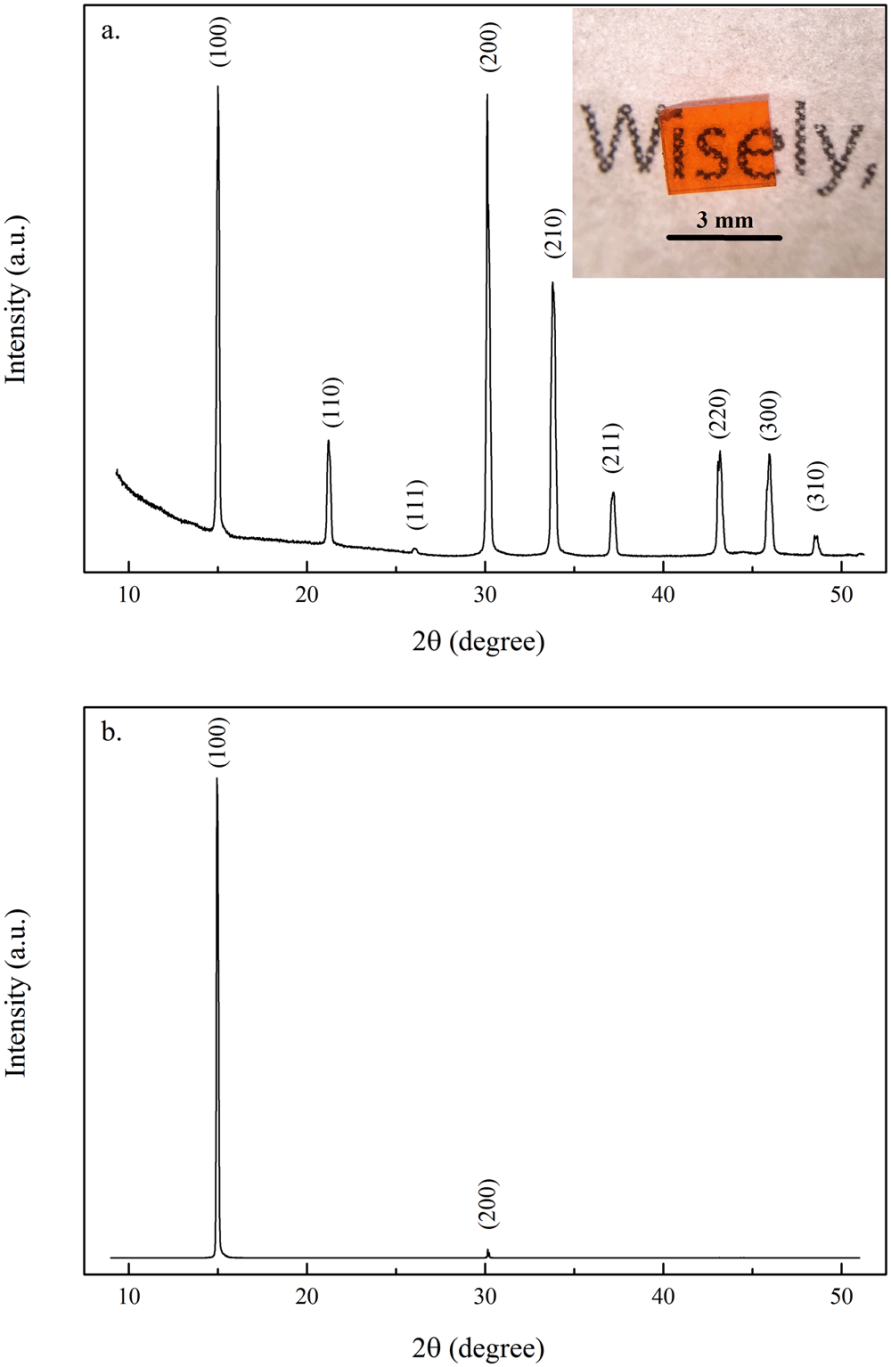
(**a**) Powder and (**b**) single-crystal XRD patterns of MAPbBr_3_ single crystal at 300 K. The inset displays an optical image of the single crystal grown by the ITC method.

The inset in Fig. 1(a) shows an optical image of a MAPbBr_3_ single crystal grown by the ITC method with dimensions of ~3.5 × 3.5 × 1.5 mm^3^. Figure 1(a) and (b) show the collected powder and single-crystal XRD patterns at 300 K, respectively. The crystal adopted the cubic ${Pm}\mathop{3}\limits^{\bar{} }m$ space group at room temperature. The measured diffraction peak positions (Fig. 1) at 15.0°, 21.21°, 26.04°, 30.12°, 33.78°, 37.15°, 43.18°, 45.93°, and 48.63° were converted into interplanar spacings, which corresponded to the (100), (110), (111), (200), (210), (211), (220), (300), and (310) crystal planes, respectively. The TEM image or selective area electron diffraction (SAED) pattern of the crystal can not be measured due to the immediate amorphization or liquidation of the material upon illumination by the energetic electron beam (Figure S1 in the Supporting information)

**Figure 2:**
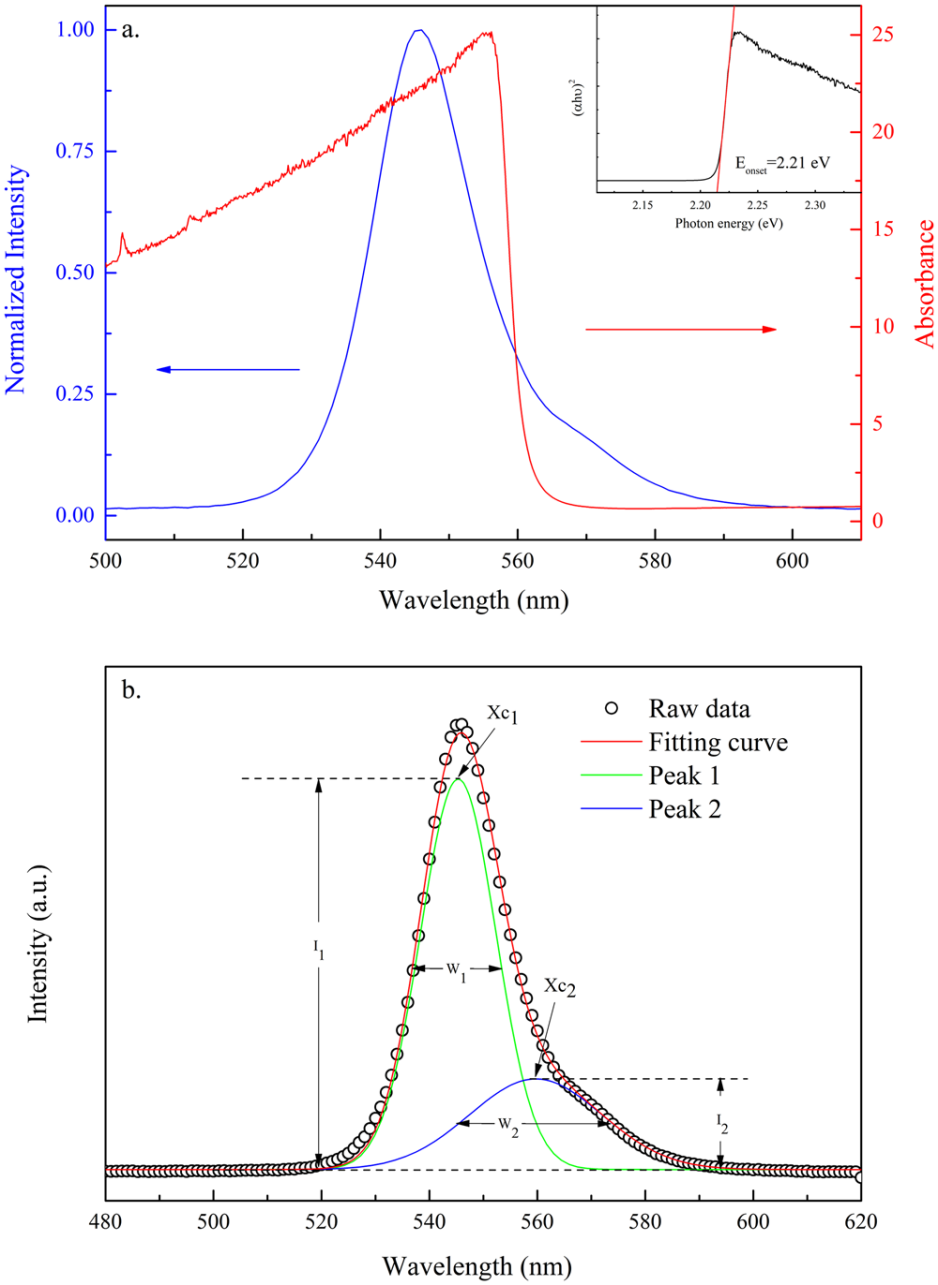
(**a**) UV-Vis absorption and PL spectra of the solution grown MAPbBr_3_ single crystal excited at 405 nm at room temperature (**b**) fitting of the PL spectrum by peaks 1 and 2. X_c1_, X_c2_, W_1_, W_2_, I_1_, and I_2_ represent the peak positions, bandwidths at FWHM, and normalized intensity of peaks 1 and 2, respectively. Inset shows the onset of the absorption at ~2.21 eV.

**Figure 3:**
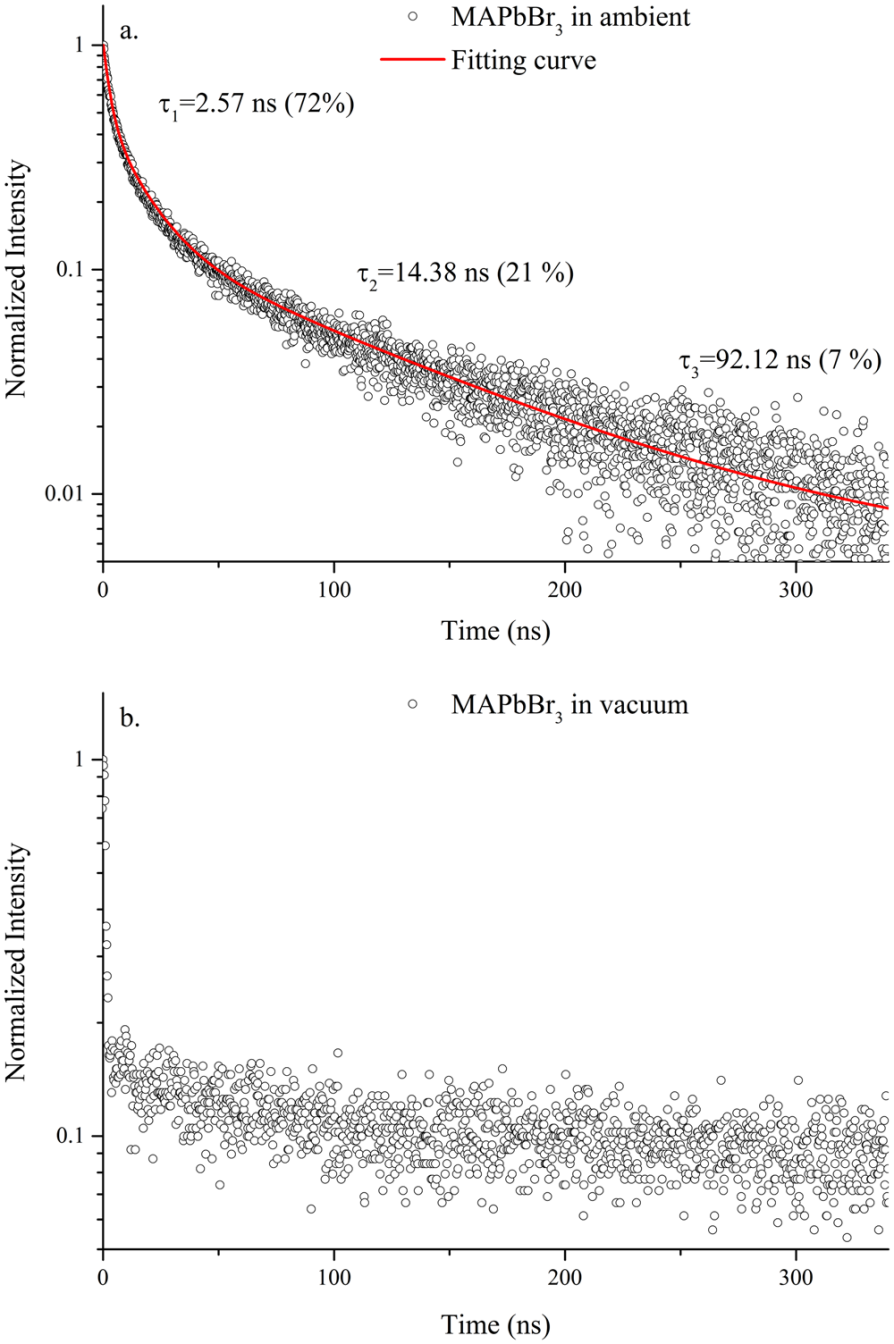
Transient PL curves recorded (**a**) under ambient conditions and (**b**) in a vacuum at an excitation wavelength of 405 nm at room temperature. The respose time of the system is ~1 ns.

The UV-Vis absorption spectrum and PL of the solution grown MAPbBr_3_ single crystal excited with a 405 nm solid-state laser at 300 K are shown in Fig. 2(a). Under ambient conditions, the excitation of the crystal resulted in a strong yellow-green fluorescence. The PL spectrum can be fitted by two emission peaks (Fig. 2(b)) and was characterized by an asymmetric line shape, which trailed to a long wavelength. The major PL peak (peak 1) above the absorption onset was at 545 nm with a linewidth of ~16 nm (FWHM) and was originated from the band-to-band transition. The lower energy peak (peak 2) at 560 nm, which had a broad linewith of 29 nm, was attributed to the recombination in trap states (Br vacancies on the crystal surface) below the optical gap^17,23,35^. The inset in Fig. 2(a) indicates that the onset of absorption occurs at ~2.21 eV, which matches with the peak position of the peak 2. We investigated the transient behavior of PL at the wavelength of peak 1 (545 nm) to understand the dynamics of photoexcited carriers in the MAPbBr_3_ single crystals. Figure 3(a) shows the transient PL curve recorded under ambient conditions at an excitation wavelength of 405 nm. The decay time of the crystal measured under ambient conditions showed an initially fast component with a lifetime of ~2.57 ns and was followed by two slow components with lifetimes of ~14.38 ns and 92.12 ns, respectively. The underlying mechanism behind the extremely long carrier decay time in the hybrid perovskite is still not clear. Our results were in good agreement with earlier studies^40,41^ on MAPbBr_3_ single crystals prepared with the same ITC method, in which they found a shorter lifetime (~1.2 ns) at the surface compared to a much longer PL decay time (~34.5 ns) in the bulk. However, our value was shorter than that reported previously^21^ in single crystal synthesized using anti-solvent vapor-assisted crystallization (AVC) strategy. The crystal prepared with the AVC method was possibly better in quality with a low trap-state density than that prepared with the ITC method.

The PL lifetime of the MAPbBr_3_ single crystal in a vacuum exceeded the system respose time, which was ~1 ns (Fig. 3(b)). This finding indicated that the MAPbBr_3_ single crystal surface was sensitive to the environment. Recent studies revealed that the interaction between hybrid perovskite materials and their environments significantly affected the material morphology itself or photostability and the optoelectronic properties^42–46^. For example, Fang *et al*.^46^ found that the PL properties of MAPbBr_3_ single crystals can be drastically modulated by exposing the single crystals to H_2_O and O_2_. Their findings was attributed to the fully and reversibly controlled surface recombination in the MAPbBr_3_ single crystal by physisorption of certain gas molecules. The difference in surface recombination velocity between passivated and unpassivated perovskite surface can be as much as two orders of magnitude^46^. Note that, even with surface passivation, the decay rate in our samples was still dominated by the fast component (72%, as shown in Fig. 3(a)) under ambient conditions. On the other hand, Tian *et al*.^47^ reported a substantial PL enhancement upon light irradiation when oxygen gas was presented in the atmosphere in MAPbI_3_ crystals. The PL lifetime increased by two orders of magnitude in the course of light treatment. Moreover, the PL enhancement was reversible by switching the illumination on and off or changing the atmosphere gas between O_2_ and N_2_. It was attributed to a photochemical reaction involving oxygen in which the trapping sites responsible for non-radiative charge recombination can be de-activated. More recently, visual evidence on compositional changes in MAPbI_3_ films upon illumination was observed which can be directly related to the photo-induced PL enhancement^48^. In contrast to the previous report by Tian *et al*.^47^, they found that oxygen may not be essential in the process of de-activating the trapping states. Nevertheless, the precise mechanism of the aforementioned photophysical bahavior is still under debate and remains an open question for the community.

**Figure 4:**
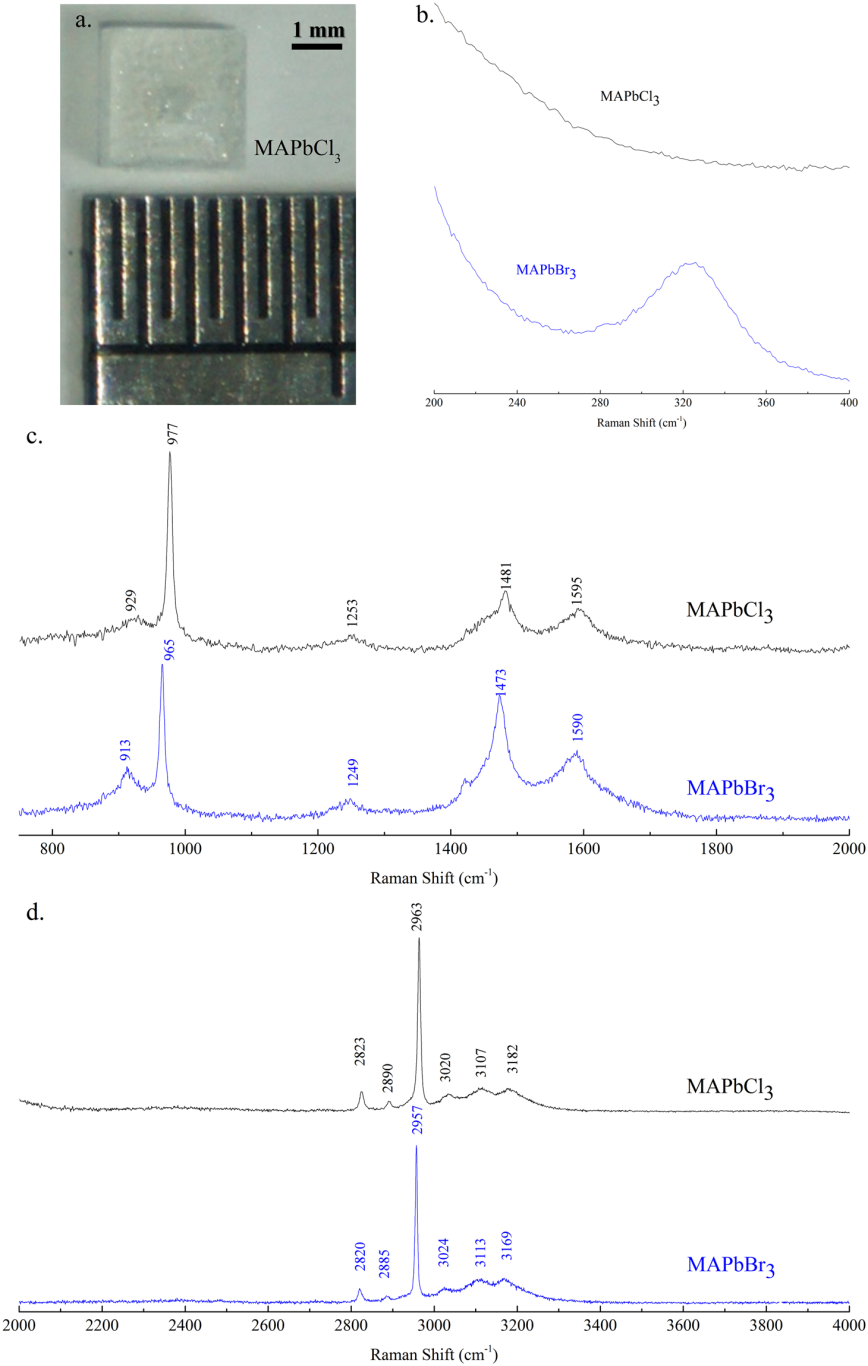
(**a**) Photo image of the as-synthesized MAPbCl_3_ single crystal and Raman spectra of MAPbBr_3_ and MAPbCl_3_ single crystals cover from (**b**) 200–400 cm^−1^ (**c**) 800–2000 cm^−1^ and (**c**) 2000–4000 cm^−1^.

The interaction between the MA^+^ cation and the PbBr_3_^−^ in the octahedral framework was investigated using Raman spectroscopy at an excitation wavelength of 632 nm to avoid the fluorescence background. Figure 4 shows the high signal-to-noise ratio of the Raman spectra from a MAPbBr_3_ single crystal that covers a wavenumber range of 200–4000 cm^−1^. The Raman spectra from a MAPbCl_3_ single crystal prepared with the ITC method are also displayed for comparison. The photo image of the as-synthesized MAPbCl_3_ single crystal is shown in Fig. 4(a). The low-frequency band at 325 cm^−1^, as shown in Fig. 4(b), is related to the restricted rotation of the MA^+^ in MAPbBr_3_. However, this peak is absent in the Raman spectrum of the MAPbCl_3_ single crystal, as shown in Fig. 4(b). The rotation mode of the MA^+^ in MAPbBr_3_ single crystal can be weakened when the Br atom is gradually replaced with a Cl atom due to the higher electronegativity strengthens of the Cl^−^^49^. In this study, the rotation mode of the MA^+^ was entirely restricted when Br was completely substituted by Cl. The intermediate- and higher-frequency bands, as shown in Fig. 4(c) and (d), were all related to the different kinds of the MA^+^ cation motions. For example, the sharp and intense bands at 913 cm^−1^ and 965 cm^−1^ were from the CH_3_NH_3_^+^ rocking and C-N stretching, respectively. The strong band at 2957 cm^−1^ was assigned to the symmetric CH_3_ stretching. Assignments and comparisons between Raman bands from MAPbBr_3_ and MAPbCl_3_ single crystals are summarized in Table 1. Replacing the Br with Cl prohibited the restricted MA^+^ rotation mode, whereas the intensity of the other bands remained almost unchanged. However, different amounts of energy shifts (either blueshift or redshift) were observed for modes, as listed in Table 1, when Br was substituted by Cl. Significant blueshifts as large as 16 cm^−1^, 12 cm^−1^, and 13 cm^−1^ were observed for the CH_3_NH_3_^+^ rocking mode, C-N stretching mode, and NH3^+^ symmetric stretching mode, respectively. These observations indicated that replacing Br with Cl changes the microenvironment of the PBX_3_^−^ framework significantly.

**Table 1:** The measured Raman shift of vibration modes, peak assignments, and shift in peak position of the MAPbBr_3_ and MAPbCl_3_ single crystal.

MAPbBr_3_ (cm^−1^)	MAPbCl_3_ (cm^−1^)	Peak assignment	Amount of shift (cm^−1^)
913	929	CH_3_NH_3_^+^ rocking	16
965	977	C–N stretching	12
1249	1253	CH_3_-NH_3_^+^ rocking	4
1473	1481	asym. NH_3_^+^ bending	8
1590	1595	NH_3_^+^ twisting	5
2820	2823	N^+^–H stretching	3
2885	2890	Asym. CH_3_ stretching	5
2957	2963	Sym. CH_3_ stretching	6
3024	3020	NH_3_^+^ Sym. stretching	−4 (red shift)
3113	3107		−6 (red shift)
3169	3182		13

The temperature-dependent crystal structure of the MAPbBr_3_ single crystal was determined on the basis of the temperature-dependent XRD measurements and temperature-dependent absorption/PL spectra. Figures S2(a) and (b) (in the supporting information) show the powder XRD and single-crystal XRD patterns of the crystal measured at 300–20 K. The crystal underwent several structural transitions at temperatures of ~220 K, ~145 K, and ~80 K, which corresponded to structural changes from cubic to tetragonal, tetragonal to orthorhombic I, and orthorhombic I to orthorhombic II, respectively. The phase transitions were accompanied by energy shifts and line shape changes in the absorption and emission spectra, as shown in the Figure S3 (in the supporting information). Temperature-dependent structure changes were categorized into three different temperature regions: 300–170 K, 170–110 K, and 140–1.5 K and are detailed as follows.

### (i) 300–170 K

**Figure 5:**
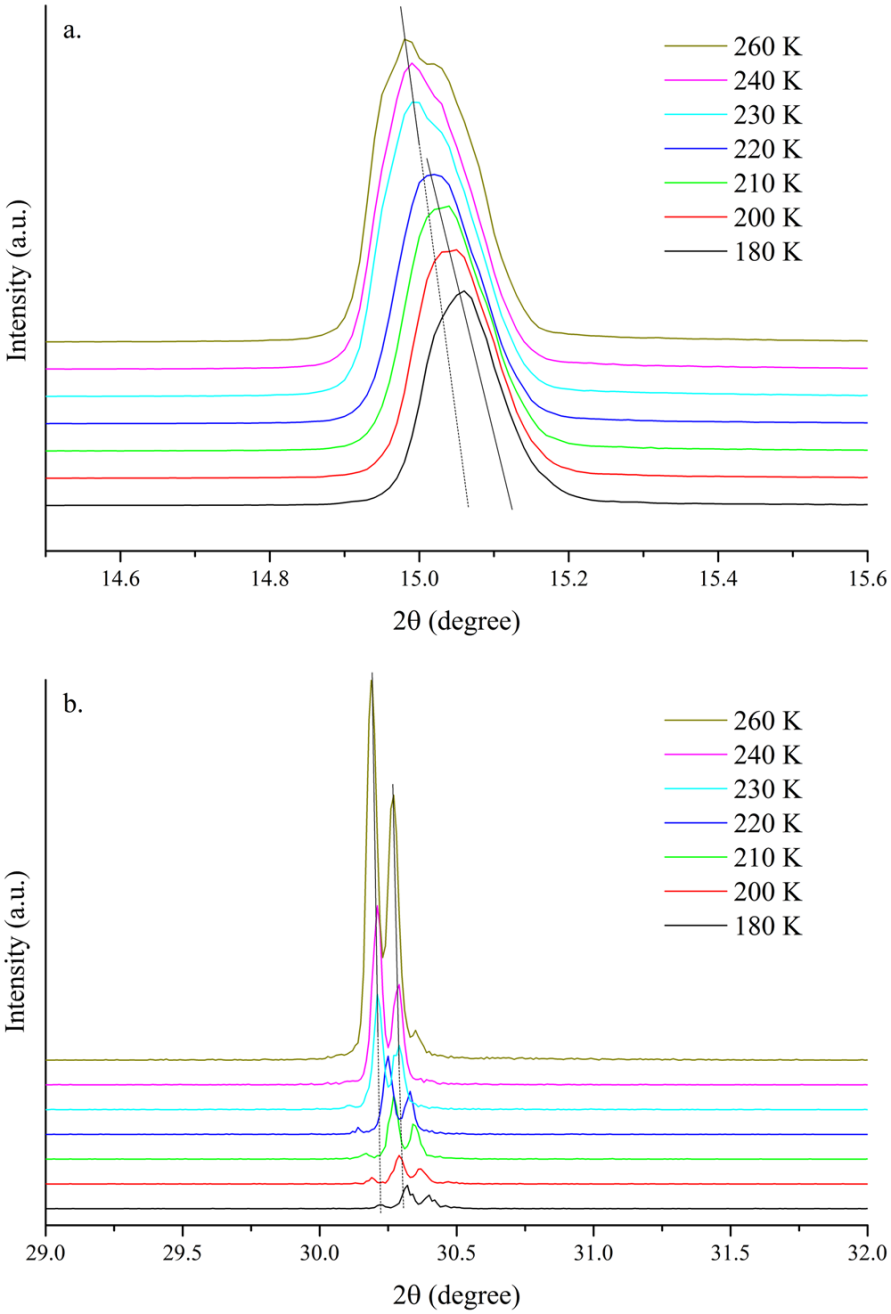
Single-crystal XRD spectra of the (100) and (200) peaks of MAPbBr_3_ single crystals at 260–180 K.

**Figure 6:**
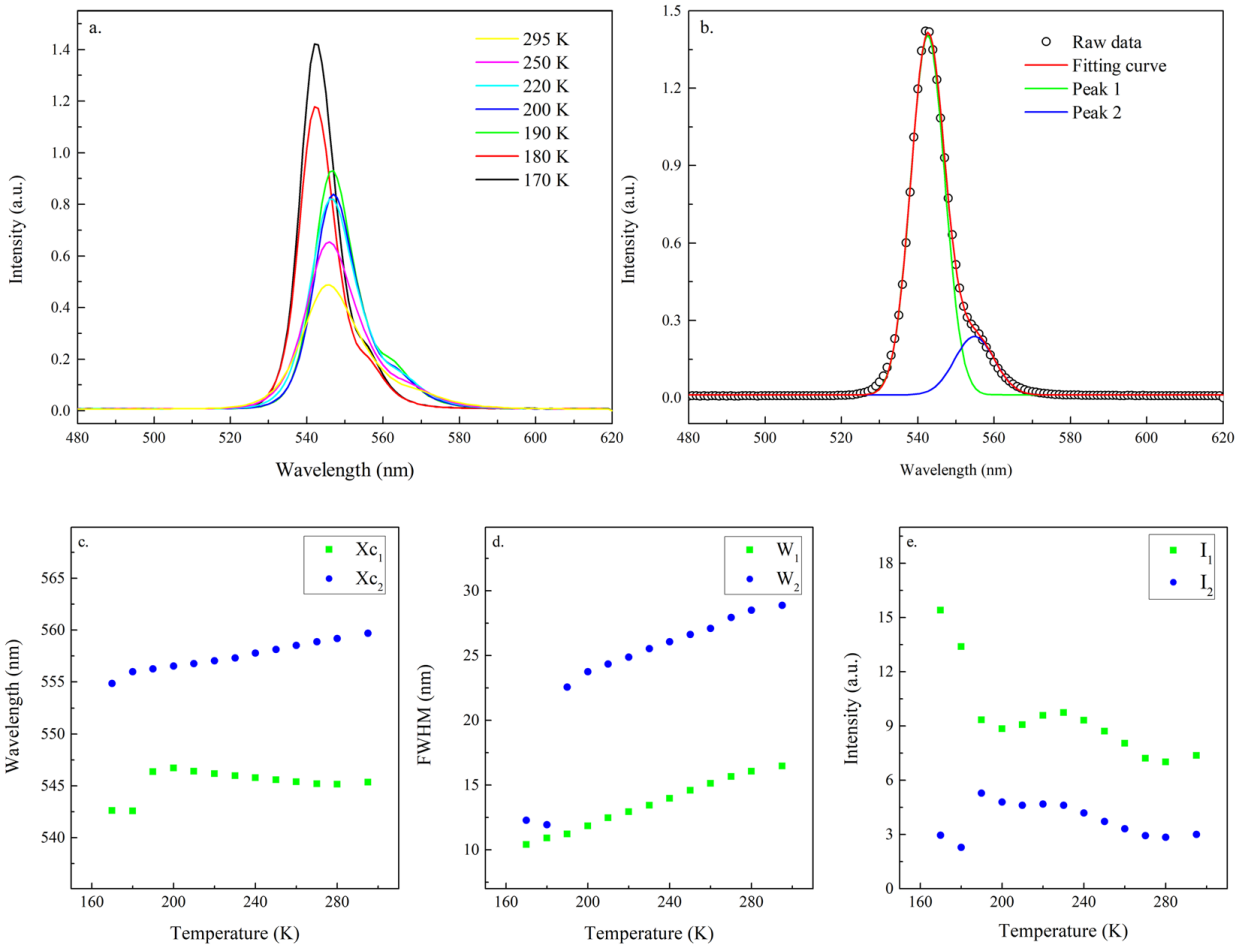
(**a**) PL spectra of MAPbBr_3_ single crystal excited at 405 nm at 295–170 K (**b**) fitting of the representative PL spectrum by peaks 1 and 2. X_c1_, X_c2_, W_1_, W_2_, I_1_, and I_2_ represent the peak positions, bandwidths at FWHM, and normalized intensity of peaks 1 and 2, respectively. (**c**,**d**,**e**) Temperature-dependent peak positions, bandwidths and intensity of peaks 1 and 2 at 295–170 K.

**Figure 7:**
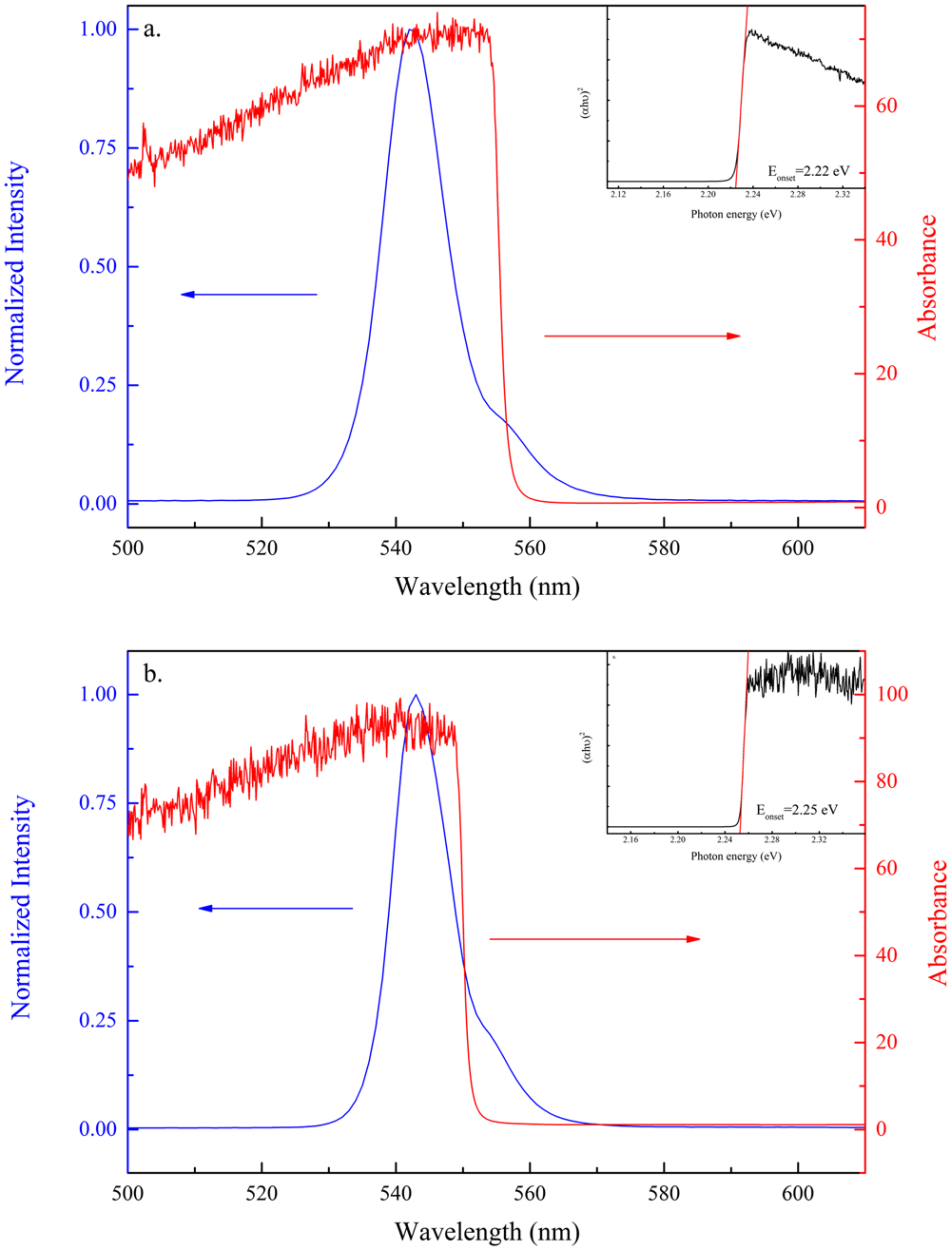
UV-Vis absorption and PL spectra of MAPbBr_3_ single crystals excited at 405 nm at (**a**) 230 K and (**b**) 180 K. The insets show the fitting of the absorption onset.

Figure 5(a) and (b) show the single-crystal diffraction spectra of crystal planes (100) and (200), respectively, when the crystal was cooled from 260 K to 180 K. Both peaks shifted toward large diffraction angles as the temperature decreased due to the lattice contraction. Moreover, both peaks showed a significant shift in the diffraction angle at approximately 220 K. The (100) peak moved from 15.02° at 220 K to 15.05° at 200 K. This change implied that a transition from the cubic to the tetragonal phase took place at ~220 K, and the phase transition was reflected in the PL and absorption spectra. As shown in Fig. 6(a), the PL line shape remained asymmetric as the crystal was cooled toward 170 K. Fitting of the PL peaks is displayed in Fig. 6(b). The PL peak position, line width, and intensity changed as a function of temperature, plotted in Fig. 6(c–e). The PL intensity of peak 1 and Peak 2 increased and the line width became narrow as the temperature decreased (Fig. 6(c) and (d)). A significant blueshift by ~16 meV in peak 1 was evident when the temperature reached 200 K. The relative intensity of peak 1 to peak 2 also increased significantly at this point (Fig. 6(e)). The change in the absorption edge was confirmed by the temperature-dependent absorption measurements (Fig. 7(a) and (b)). The fitted absorption onset shifted from 2.22 eV at 230 K to 2.25 eV at 180 K. The difference in the critical temperature of phase transition by XRD studies (at ~210 K) and of the electronic structure change by optical studies (at ~190 K) is due to larger temperature fluctuation induced by the electrical heating element in the liquid helium dilution refrigerator at higher temperature. The structural change from cubic to tetragonal was mainly due to the rotation of the PbBr_6_^4+^ octahedron around the c-axis (Figure S4 in the supporting information), which also leads to a larger bandgap^24,50–52^.

### (ii) 170 K–110 K

**Figure 8:**
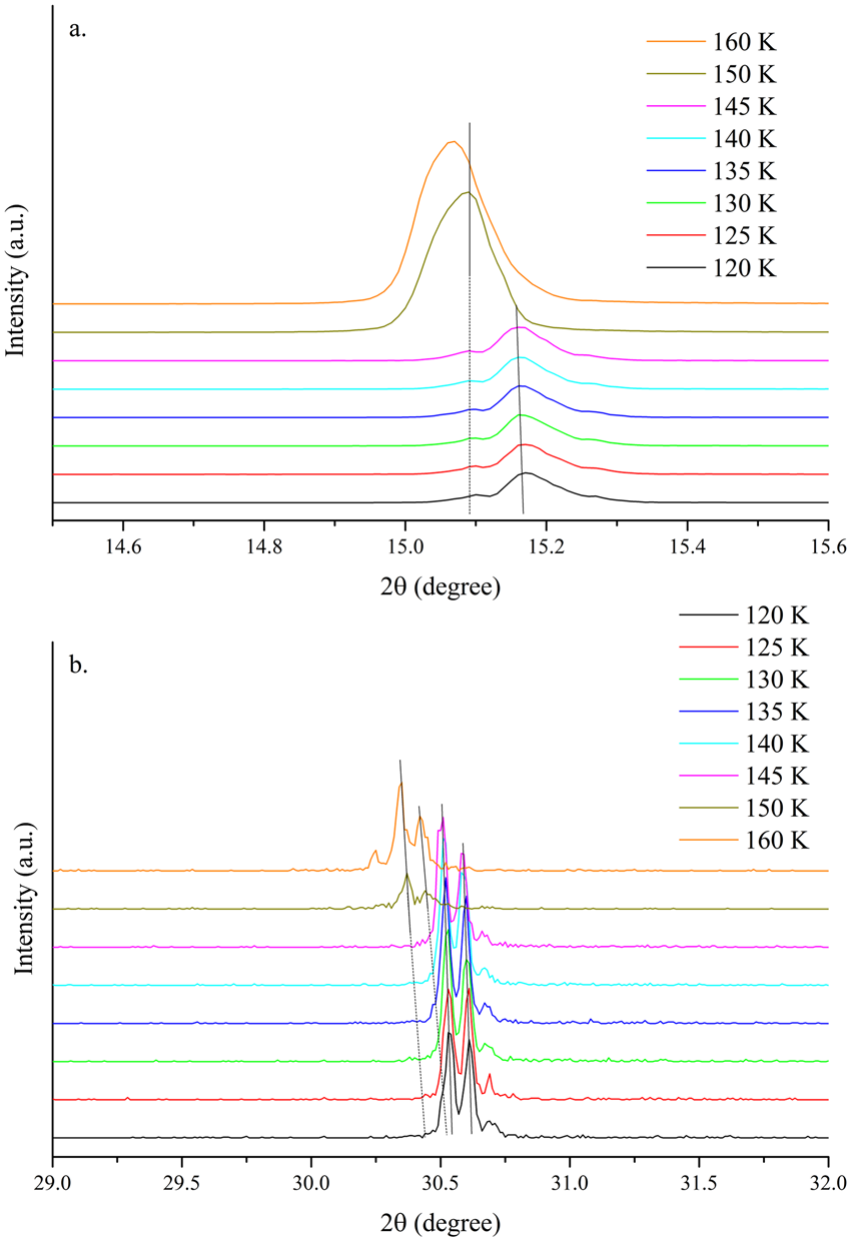
Single-crystal XRD spectra of (100) and (200) peaks of MAPbBr_3_ single crystals at 160–120 K.

**Figure 9:**
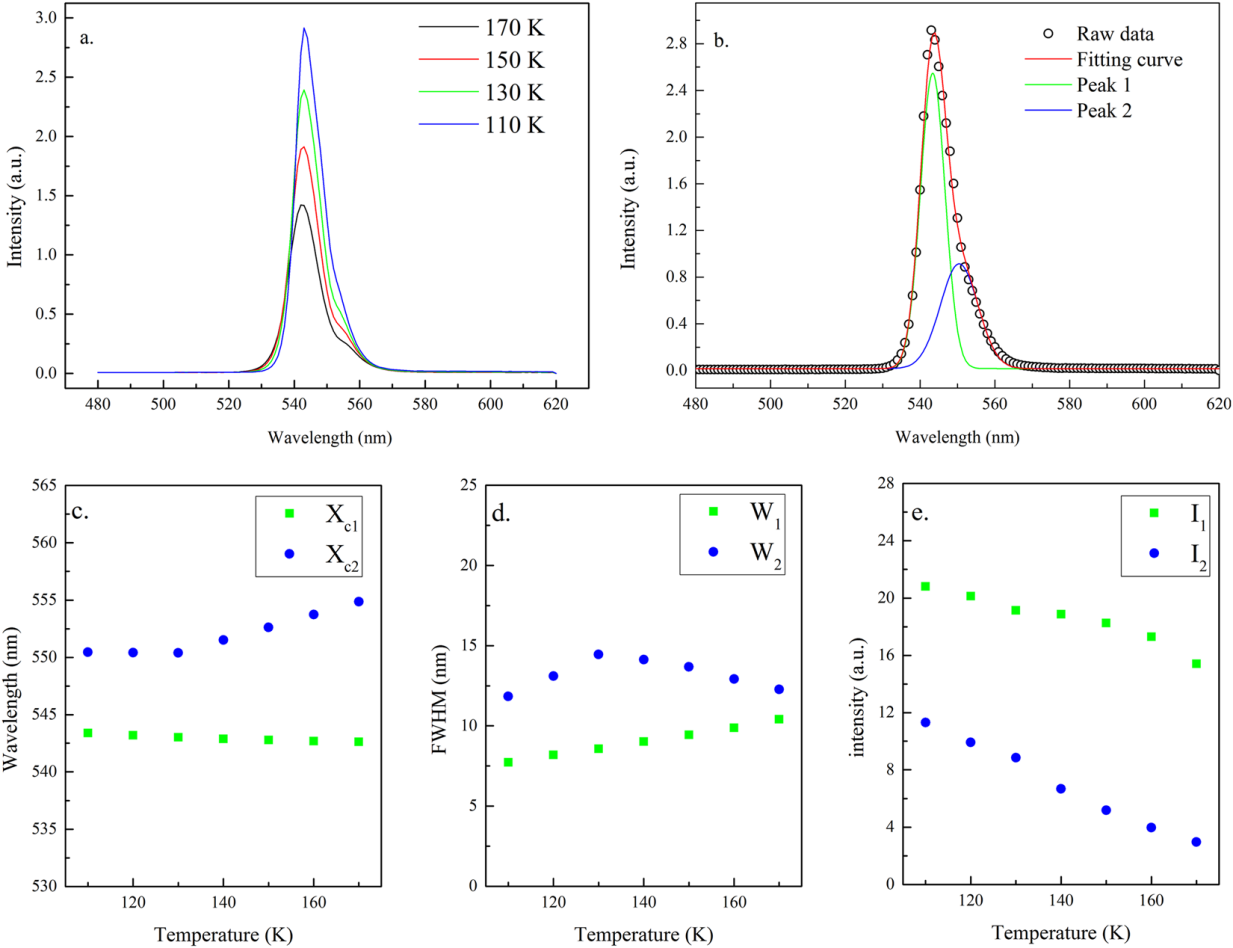
(**a**) PL spectra of MAPbBr_3_ single crystals excited at 405 nm at 170–110 K (**b**) fitting of the representative PL spectrum by peaks 1 and 2. X_c1_, X_c2_, W_1_, W_2_, I_1_, and I_2_ represent the peak positions, bandwidths at FWHM, and normalized intensity of peaks 1 and 2, respectively. (**c,d,e**) Temperature-dependent peak positions, bandwidths and intensity of peaks 1 and 2 at 170–110 K.

As the temperature dropped from 170 K to 110 K, the MAPbBr_3_ crystal underwent a second phase transition, which was from tetragonal to orthorhombic I, as shown by the larger diffraction angle shift at ~145 K (Fig. 8(a) and (b)). The phase transition was triggered when the PbBr_6_^4+^ octahedron tilted out of the ab plane. However, contrary to the previous temperature stage, some tetragonal signals remained after the phase transition (see XRD spectrum in Fig. 8(a)). We considered the possibility of the coexistence of the crystallographic phases of the tetragonal and orthorhombic stages. Moreover, coexisting crystallographic phases have been reported for some inorganic perovskite materials with mixed or pure compositions^53–58^. Unequal thermal expansion or spontaneous changes in the in-plane lattice constants during the phase transition may produce a significant amount of strain on the crystal thereby leading to phase coexistence^24^; however, lattice-mismatch can not be accounted for the coexisting crystallographic phase. The behavior of the PL spectra in this temperature stage was similar to that in the previous temperature stage, except that the energy, linewidth, and relative intensity changes were not significant (Fig. 9).

### (iii) 140 K–1.5 K

**Figure 10:**
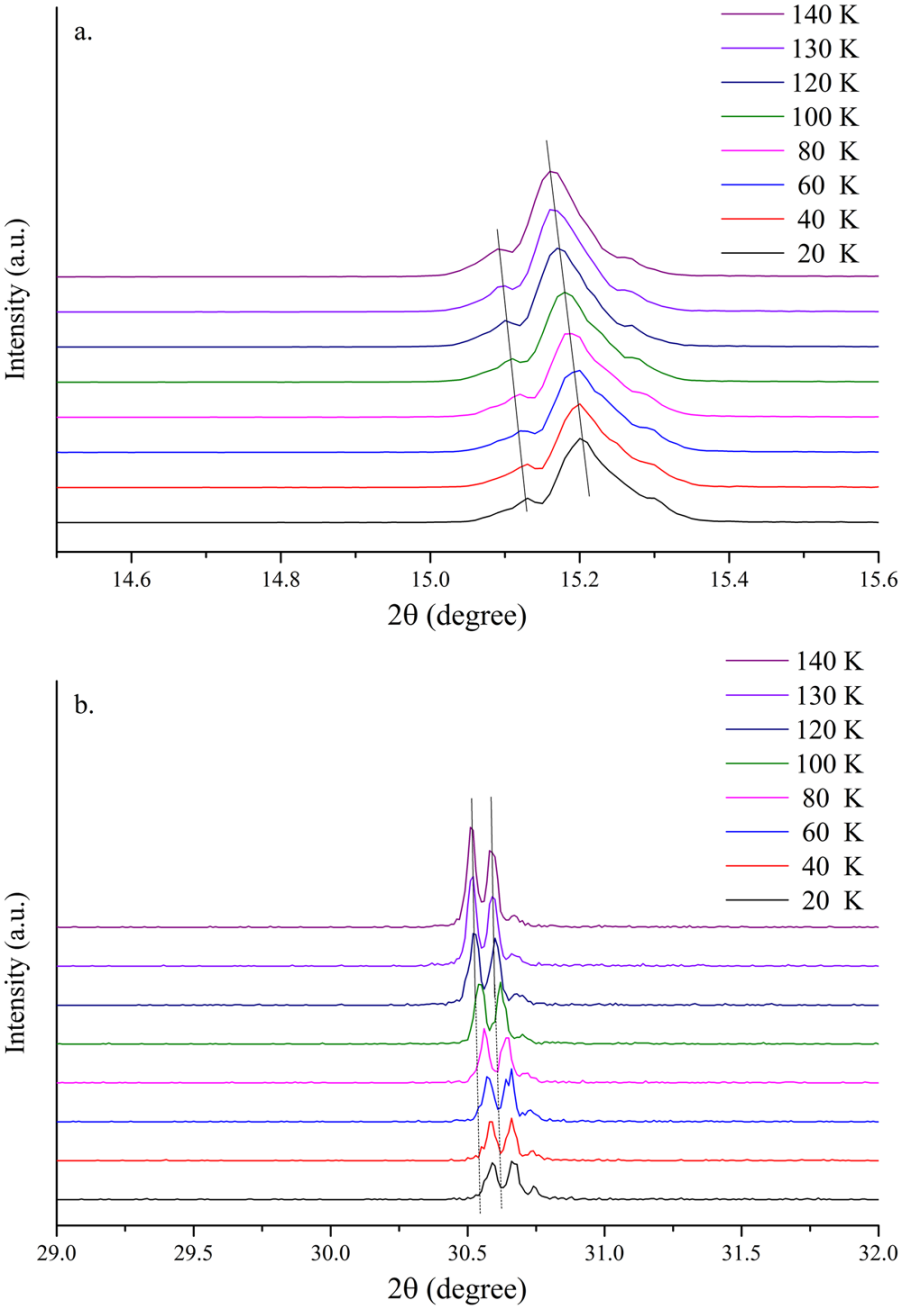
Single-crystal XRD spectra of (100) and (200) peaks of MAPbBr_3_ single crystal at 140–20 K.

**Figure 11:**
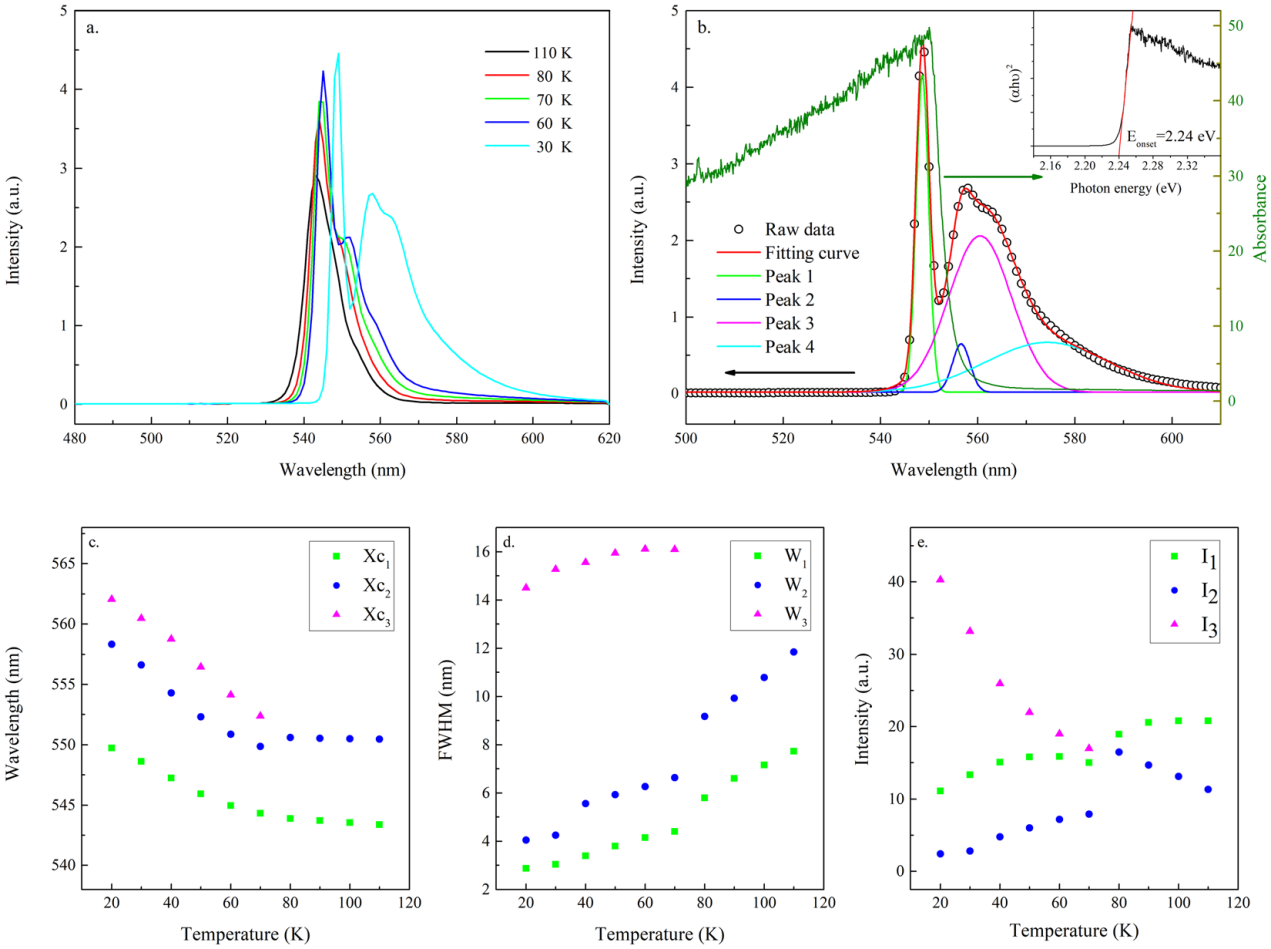
(**a**) PL spectra of MAPbBr_3_ single crystals excited at 405 nm at 110–30 K (**b**) fitting of the representative PL spectrum by peaks 1, 2, 3, and 4. X_c1_, X_c2_, X_c3_, W_1_, W_2_, W_3_, I_1_, I_2_, and I_3_ are the peak positions, bandwidths, and intensity of peaks 1, 2, and 3, respectively. (**c**,**d**,**e**) Temperature-dependent peak positions, bandwidths, and intensity of peaks 1, 2, and 3 at 110–30 K. The inset in (**b**) shows the fitting of the absorption onset at 30 K.

**Figure 12:**
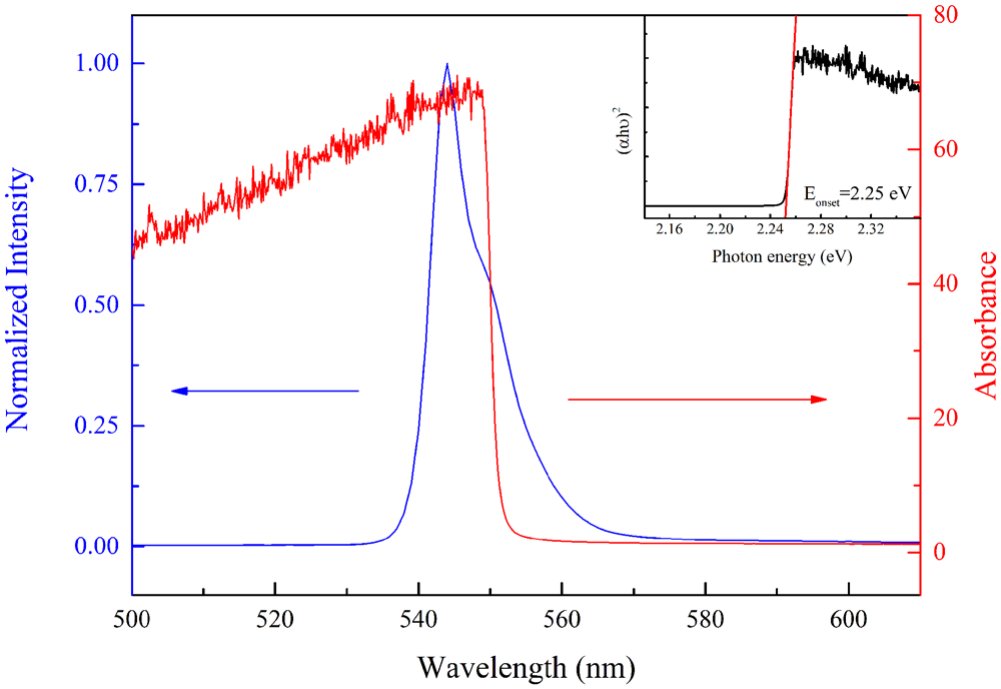
UV-Vis absorption and PL spectra of MAPbBr_3_ single crystals excited at 405 nm at 80 K. Inset shows the fitting of the absorption onset at ~2.25 eV.

Figure 10 shows the temperature-dependent single-crystal XRD spectra obtained from 140–20 K. A large diffraction angle shift occurred at ~80 K for the peak of (200) plane. Dramatic changes in the PL line shape and energy positions took place at the same temperature (Fig. 11(a) and (b)). Contrary to the two previous temperature stages, the representative PL spectrum measured at 30 K in this stage can be fitted with four emission peaks (Fig. 11(b)). The emission peaks 3 and 4 only appeared when the temperature was below 80 K. The absorption spectrum measured at 80 K (Fig. 12) gave a bandgap energy of ~2.25 eV, which was approximately the same as that at 180 K (Fig. 7(b)). Therefore, emission peaks 1 and 2 can be attributed to the transitions of the coexisting tetragonal phase and Br vacancy, respectively. The appearance of the emission peak 3 below 80 K was possibly due to the secondary phase transition from orthorhombic I to orthorhombic II, which was caused by the increased tilting angle of the PbBr_6_^4+^ octahedron with respect to the ab plane (Figure S4 in the supporting information). The broad emission line of peak 4 is attributed to defect states associated with the orthorhombic II phase. Surprisingly, all three emission peaks (peaks 1, 2, and 3) were redshifted when the crystal was cooled from 80 K to 20 K (Fig. 11(c)). Further experiments are required to elucidate this phenomenon. Note that the intensity of peak 3 increased rapidly while the intensity of peaks 1 and 2 slowly decayed at a temperature below 80 K (Fig. 11(e)), which was also an indication of the phase transition from orthorhombic I to orthorhombic II. Figure S4 in the supporting information summarizes the structural changes throughout the cooling process through a schematic of the 3D MAPbBr_3_ crystal structure at different phases.

**Figure 13:**
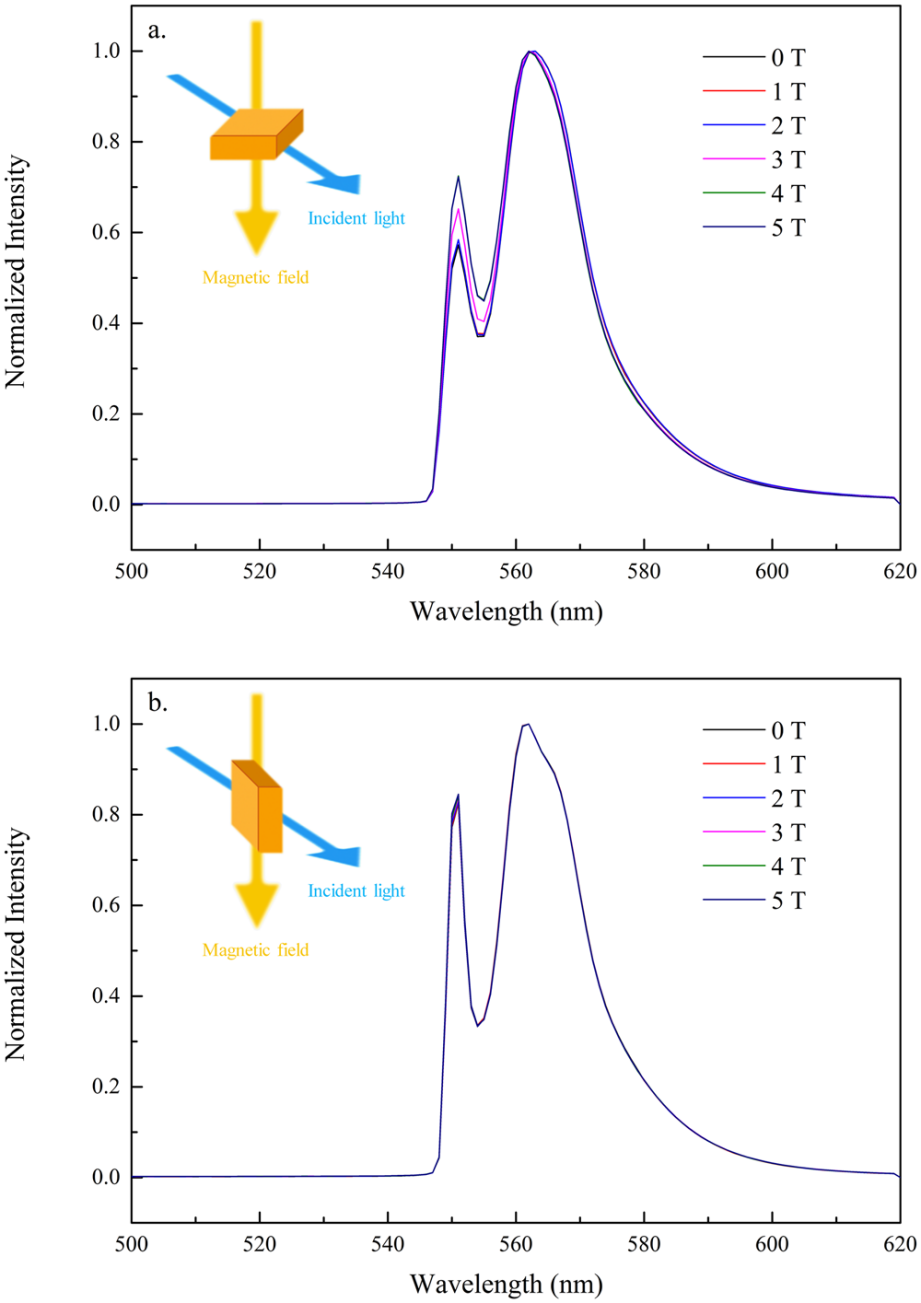
Magneto-PL spectra as a function of the magnetic field in Faraday geometry with the field applied (**a**) parallel and (**b**) perpendicular to the (100) direction at 1.5 K and scanned magnetic field up to 5 T.

Finally, we investigated the excitonic properties from the MAPbBr_3_ single crystal by cooling the crystal to 1.5 K in a refrigerator and scanning a magnetic field up to 5 T at an excitation wavelength of 405 nm. Figure 13 shows the magneto-PL spectra as a function of the magnetic field in Faraday geometry, with the field applied parallel and perpendicular to the (100) direction. This test aimed to determine if the emission peaks in the PL spectra possessed excitonic properties. The data indicated that the emission lines did not exhibit diamagnetic shifting or energy splitting even at a magnetic field of 5 T. This finding implied that those transitions are unlikely to be excitonic even at a temperature as low as 1.5 K.

## Conclusions

The structural and photophysical properties of single-crystalline MAPbBr_3_ were investigated using temperature-dependent XRD and various spectroscopy techniques. The crystal underwent three phase transitions: from cubic to tetragonal, tetragonal to orthorhombic I, and orthorhombic I to orthorhombic II as temperature was lowered from 300 K to 20 K. The surface of the crystal was sensitive to the environment upon light illumination. However, the precise mechanism is still controversial and remains an on going question for the community. The intensity of the restricted rotation mode of the MA^+^ in MAPbBr_3_ was diminished upon replacing Br with an atom with high electrongegativity, such as Cl. Moreover, substituting Br with Cl also led to different amount of band shift in the Raman spectra. The magneto-PL studies implied that emissions from the crystal exhibited neither free nor bound excitonic nature even at a very low temperature.

## Electronic supplementary material

Supplementary Information
