# Supplementary material for: Structural and Photophysical Properties of Methylammonium Lead Tribromide (MAPbBr3) Single Crystals
Source: Sci Rep. 2017 Oct 20;7:13643. doi: 10.1038/s41598-017-13571-1 (PMC5651898; doi:10.1038/s41598-017-13571-1)
Supplement: Supplementary file 1 — Supplementary Information [file 41598_2017_13571_MOESM1_ESM.pdf]

Supporting Information

Structural and Photophysical Properties of Methylammonium Lead Tribromide ($MAPbBr_3$) Single Crystals

Kai-Hung Wang[1], *Liang-Chen Li*[3], *Muthaiah Shellaiah*[1], *and Kien Wen Sun*[1,2,3,*]

[1]Department of Applied Chemistry, National Chiao Tung University, 1001 University Road, Hsinchu 30010, Taiwan

[2]Department of Electronics Engineering, National Chiao Tung University, 1001 University Road, Hsinchu 30010, Taiwan

[3]Center for Nano Science and Technology, National Chiao Tung University, Hsinchu 30010, Taiwan.

Table of contents:

SEM and TEM images of $MAPbBr_3$ single crystals **(S3)**

XRD patterns of $MAPbBr_3$ single crystals measured at 300 - 20 K **(S4)**

Contour mapping of the the PL spectra **(S5)**

Illustraion of the structural changes at different temperature stages **(S6)**

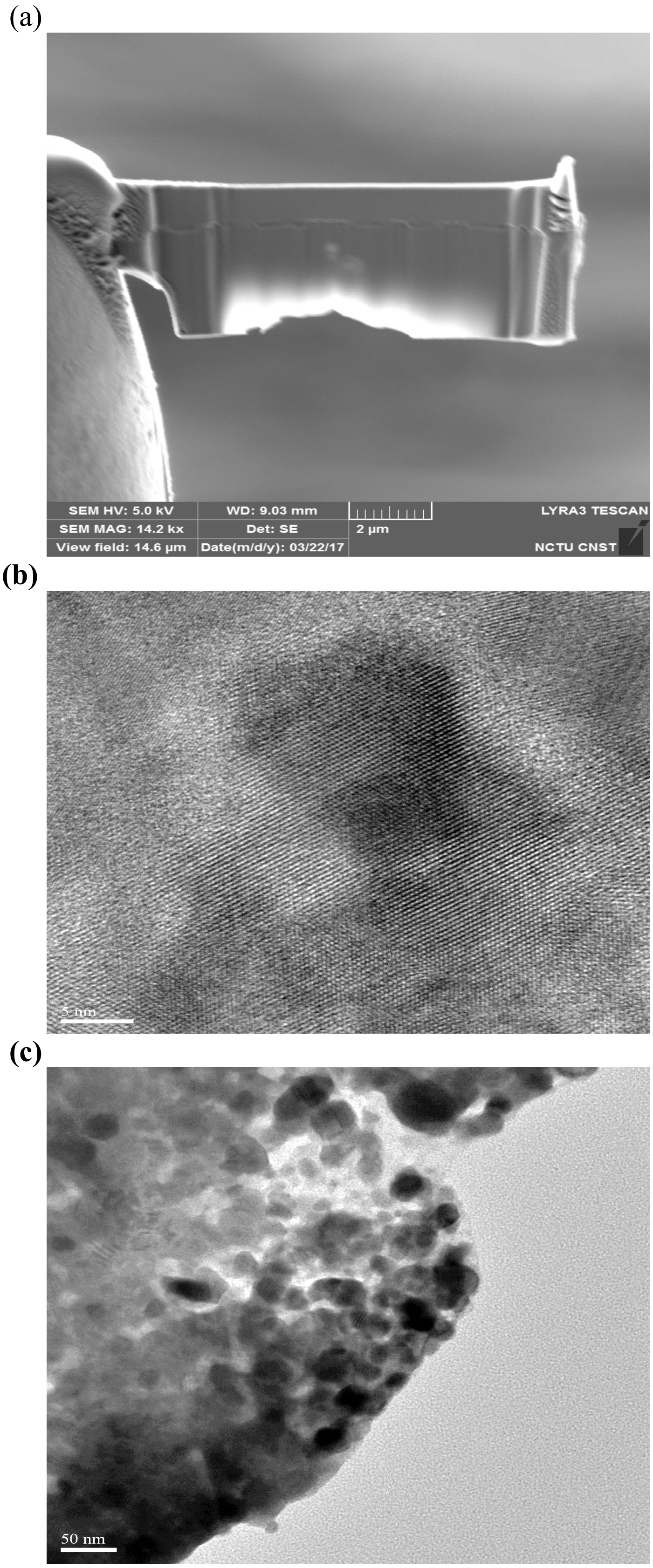

Figure S1. (a) SEM image of a thin slice cut from $MAPbBr_3$ single crystals using focus ion beam (b) TEM image of of $MAPbBr_3$ single crystal (c) TEM image of $MAPbBr_3$ single crystal powder. Amorphization or liquidation of the samples immediately occurred during the observation.

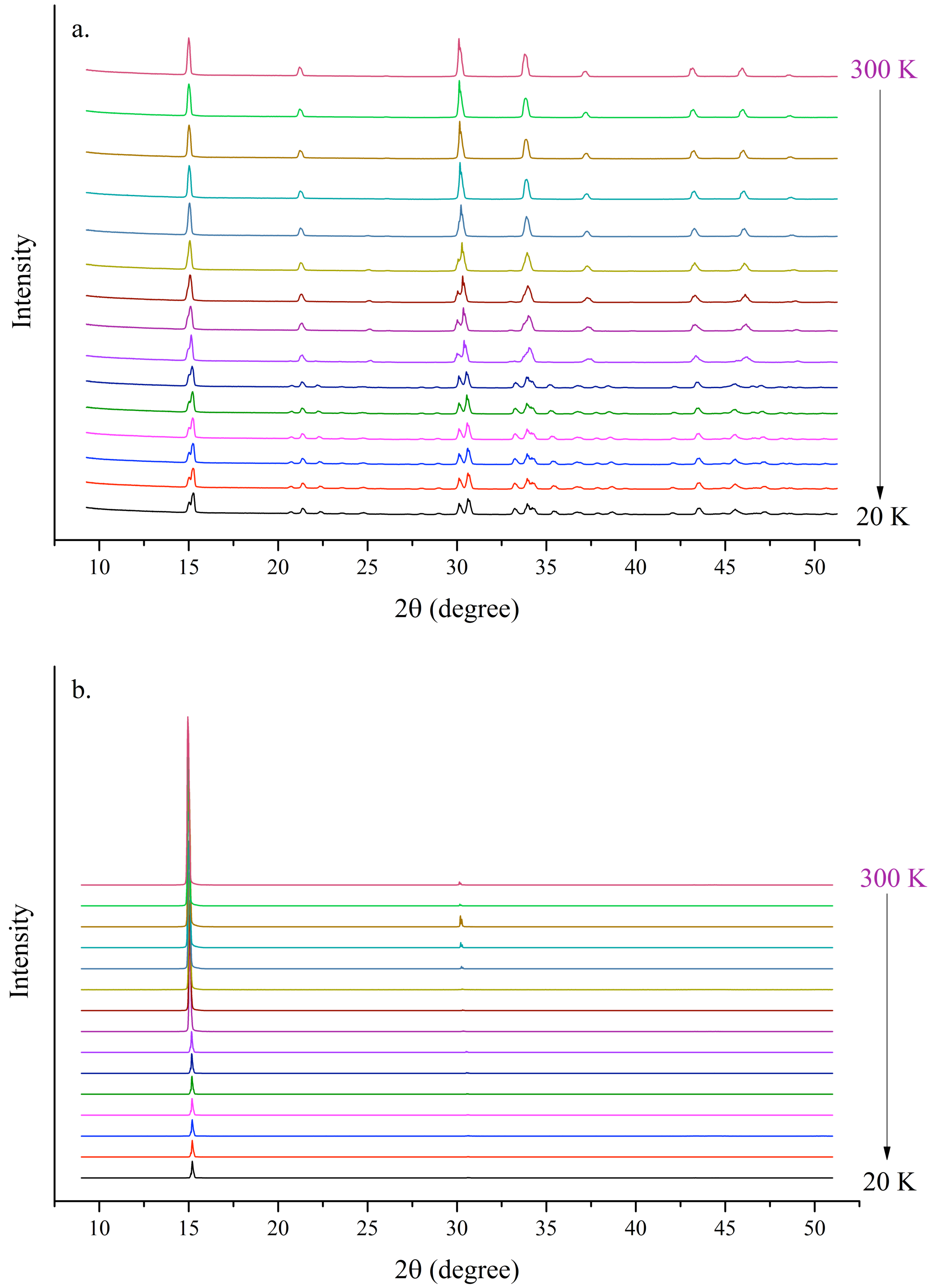

Figure S2. (a) The powder and (b) single-crystal XRD patterns of $MAPbBr_3$ single crystals measured at 300 - 20 K. The doublet structure in the (200) peak is due to the slight difference in the diffraction angle between the Kα1 and Kα2 lines.

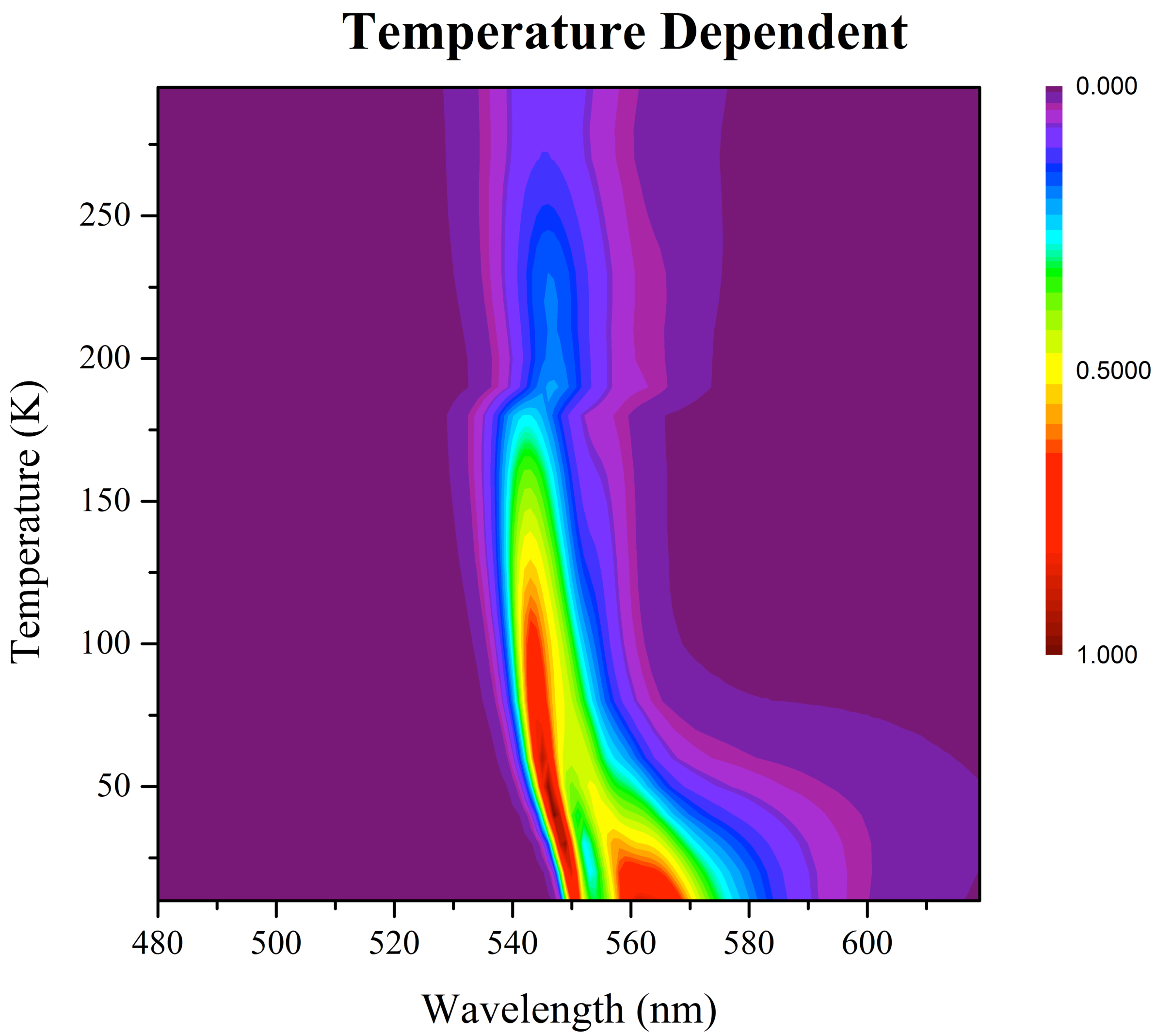

Figure S3. Contour mapping of the the PL spectra of $MAPbBr_3$ single crystals measured at 300 - 20 K.

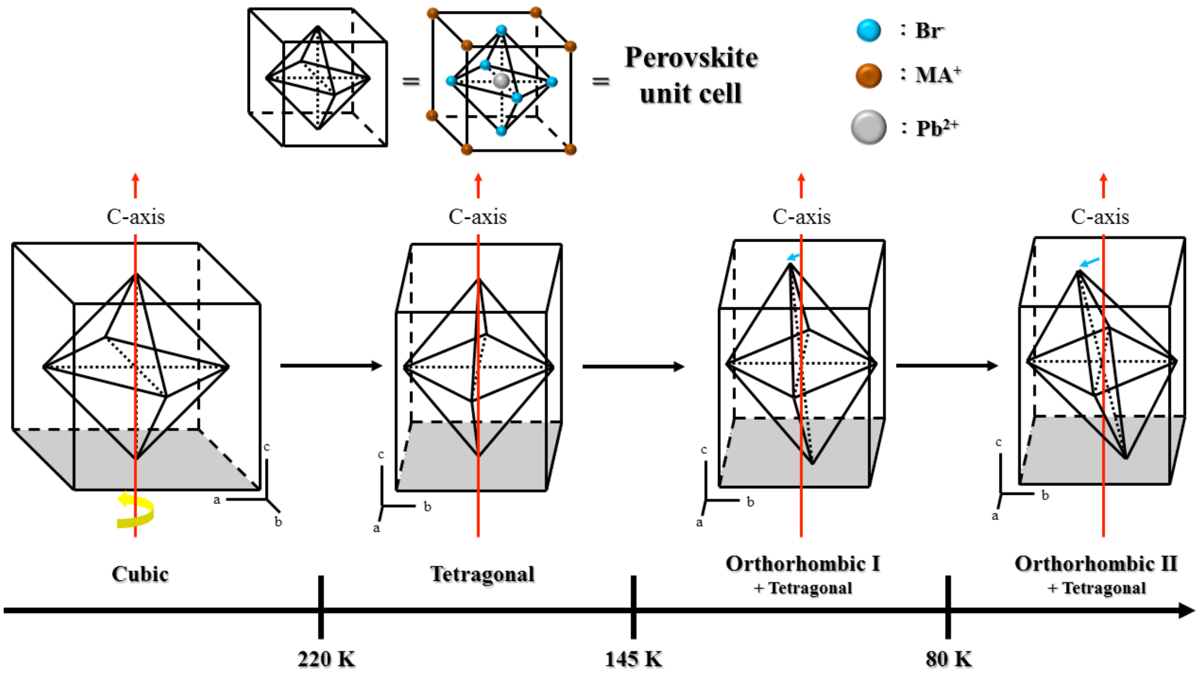

Figure S4. The structural changes at different temperature stages throughout the cooling process are displayed through a schematic of the 3D $MAPbBr_3$ crystal structure.
